# Long term methionine restriction: Influence on gut microbiome and metabolic characteristics

**DOI:** 10.1111/acel.14051

**Published:** 2024-01-26

**Authors:** Akash Nagarajan, Alexander Tate Lasher, Casey D. Morrow, Liou Y. Sun

**Affiliations:** ^1^ Department of Biology University of Alabama at Birmingham Birmingham Alabama USA; ^2^ Department of Cell, Developmental and Integrative Biology University of Alabama at Birmingham Birmingham Alabama USA

**Keywords:** aging, long‐term methionine restriction, metabolism, microbiome

## Abstract

The Methionine restriction (MR) diet has been shown to delay aging and extend lifespan in various model organisms. However, the long‐term effects of MR diet on the gut microbiome composition remain unclear. To study this, male mice were started on MR and control diet regimens at 6 months and continued until 22 months of age. MR mice have reduced body weight, fat mass percentage, and bone mineral density while having increased lean mass percentage. MR mice also have increased insulin sensitivity along with increasing indirect calorimetry markers such as energy expenditure, oxygen consumption, carbon dioxide production, and glucose oxidation. Fecal samples were collected at 1 week, 18 weeks, and 57 weeks after the diet onset for 16S rRNA amplicon sequencing to study the gut microbiome composition. Alpha and beta diversity metrics detected changes occurring due to the timepoint variable, but no changes were detected due to the diet variable. The results from LEfSe analysis surprisingly showed that more bacterial taxa changes were linked to age rather than diet. Interestingly, we found that the long‐term MR diet feeding induced smaller changes compared to short‐term feeding. Specific taxa changes due to the diet were observed at the 1 or 18‐week time points, including *Ileibacterium, Odoribacter, Lachnoclostridium, Marinifilaceae*, and *Lactobacillaceae*. Furthermore, there were consistent aging‐associated changes across both groups, with an increase in *Ileibacterium* and *Erysipelotrichaceae* with age, while *Eubacterium_coprostanoligenes_group, Ruminococcaceae, Peptococcaceae*, and *Peptococcus* decreased with age.

## Graphical abstract

A long‐term Methionine restriction diet in mice had less impact on the gut microbiome compared to short‐term feeding, with aging showing more significant effects on bacterial taxa. Specific dietary changes in bacterial taxa occurred at 1 or 18‐week intervals, while consistent age‐related changes were noted in certain taxa across both diet groups.

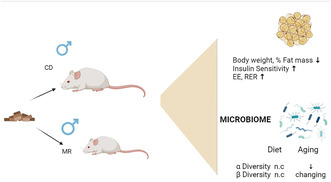

**Abbreviation**
MR: methionine restriction

## 1 INTRODUCTION

Methionine is an essential amino acid and it is required to be ingested through dietary sources. The restriction of dietary methionine below a certain threshold level in model organisms leads to health benefits and increased longevity. Methionine restriction (MR) was first shown to delay aging and increase lifespan in rats and the phenomenon was distinct from caloric restriction (CR) as the animals had equal or increased food consumption (Orentreich et al., 1993). Subsequent studies showed that MR extends mouse lifespan when initiated at 6 weeks old and when initiated in adulthood at 12 months old (Sun et al., 2009). Concurrently MR diet feeding was also found to increase longevity in another common model organism *Drosophila Melanogaster* (Lee et al., 2014). The increased longevity of wild‐type mice under the MR diet has been replicated in different labs and in different cohorts (Brown‐Borg et al., 2014). MR diet feeding was also found to boost healthspan and lifespan in progeroid mice (Bárcena et al., 2018). It has also been found that intact growth hormone signaling is required for methionine sensing and the effects of MR to be observed in mice (Brown‐Borg et al., 2014). While no direct experimental studies exist in humans, MR has also been shown to confer health benefits to cancer patients (Hoffman, 2019). The benefits of MR reported across species indicate that the mechanism(s) for its effect on lifespan are evolutionarily conserved, and thus represent a promising avenue for understanding the regulation of lifespan.

In the past two decades, the gut microbiome has been associated with many diseases. Diet plays a big role in affecting the gut microbiome composition (Turnbaugh et al., 2009), influencing bodily function, and the incidence of disease (Murphy et al., 2015). These broad influences of diet necessitate close examination of overall and specific gut microbiome composition changes occurring due to dietary manipulation. High‐fat diet feeding leads to bodily decline resulting in metabolic deficits. Previous studies have found that a high‐fat diet changes the gut microbiome composition (Murphy et al., 2015). CR in mice significantly changes the microbiome and microbiome transplantation from CR mice to germ‐free mice leads to metabolic benefits along with fat browning (Fabbiano et al., 2018). A protein‐restricted (PR) diet leads increased lifespan in mice (Solon‐Biet et al., 2014) and in other invertebrate organisms, and short‐term PR in mice leads to changes in the microbiome composition (Pak et al., 2019). Recent studies have shown that short‐term methionine‐restricted diet feeding also leads to distinct changes in the microbiota of lab mice (Yang et al., 2019) (Nichenametla et al., 2021). A study in C57BL/6 mice found that short‐term MR leads to changes in the Family level with an increase in *Bateroidaceae* and *Verrucoccaceae* and a decrease in *Ruminococcaceae* observed (Wallis et al., 2020).

Most of the dietary studies have investigated the short‐term effects on the microbiome, with the long‐term effects of MR eluding investigation. In the present study, we investigate the metabolic consequences of long‐term MR in‐vivo along with a longitudinal assessment of microbiome composition. We show that long‐term MR confers benefits to glucose homeostasis and alterations in metabolism, however, the changes in microbiome composition are largely transient.

## 2 METHODS

### 2.1 Animals and diet

Wild‐type C57BL/6 mice were bred with wild‐type BALB/c mice to produce mice on a heterogeneous genetic background to reduce artifactual findings due to using inbred mice. Subsequence generations of mice were used for experiments. The experimental mice were fed a standard chow diet (NIH 31, Purina Lab Diet) until 6 months of age. At 6 months of age, the mice were randomly divided into two groups with one group receiving a 0.16% methionine restricted diet (TD.10230, Adj. Methionine diet 0.16%, no Cys; Envigo Teklad diets, Madison WI) and the other group a 1.3% methionine diet (TD.10232, Adj. Methionine diet 1.3%, no Cys; Envigo Teklad diets, Madison WI). Weekly body weight was collected during the first 11 weeks of diet change and intermittent data on week 19, 21, 25, and 26. Food consumption data was collected for a period of week 6 to week 11 from one cage of animals per group. All experimental protocols utilizing live animals were approved by the University of Alabama at Birmingham Institutional Animal Care and Use Committee.

### 2.2 Body composition

DXA scans were performed in 18‐month‐old mice (48 weeks after diet onset) by the UAB Small Animal Phenotyping Core as previously described (Icyuz et al., 2020) using a GE Lunar PIXImus DXA machine. Briefly, isoflurane anesthetized mice were placed inside the imaging machine in a prostrate position. The head region was excluded from the scans. Data obtained included fat mass, lean mass, bone mineral density (BMD) and bone mineral content (BMC).

### 2.3 Glucose and insulin tolerance tests

Glucose tolerance testing (GTT) and insulin tolerance testing (ITT) were performed in 12‐month‐old mice (24 weeks after diet onset) following an overnight (GTT) or 4‐h (ITT) fast. Mice were administered 1 g/kg glucose or 1 IU/kg porcine insulin (Sigma‐Aldrich) via intraperitoneal (IP) injection. Blood glucose measurements were taken from a tail nick using a handheld blood glucometer (AgaMatrix PRESTO) immediately prior to injection (“minute 0”) and at the time points indicated in figures.

### 2.4 Indirect calorimetry

The comprehension lab animal monitoring system (Oxymax‐CLAMS; Columbus Instruments Co., Columbus, OH) was utilized to perform indirect calorimetry experiments. This system utilized zirconia and infrared sensors to detect oxygen (O2) and carbon dioxide (CO2) levels from individually housed mice with ad‐libitum access to their assigned diet and standard drinking water. To eliminate artifactual findings resulting from the home‐cage to indirect calorimetry cage environment change, all animals were subjected to three independent indirect calorimetry experiments with different acclimation and data collection periods over a period of 4 months. The first experiment was 48 h, with the first 24 h being considered acclimation where data was not analyzed. The second experiment was carried out over 96 h, the first 48 of which were considered acclimation. The final experiment was carried out over 196 h (7 days) with the first 48 h considered acclimation. A 1‐month recovery period was allowed between all experiments. Data collection occurred once every 9 min for each respiratory chamber. The collected data were averaged into hourly values for a single 24‐h period for analysis. Respiratory exchange ratio (RER) and energy expenditure (EE) were calculated as previously described (Icyuz et al., 2020). Fat oxidation (FOX) and glucose oxidation (GOX) were calculated as FOX = (1.96*VO2)−(1.96*VCO2) and GOX = (4.57*VCO2)−(3.23*VO2). Indirect calorimetry was performed on 18‐22‐month‐old mice (about 48 to 64 weeks after diet onset).

### 2.5 16S rRNA microbiome sequencing

Fecal samples were collected at three different time points: 1 week, 18 weeks, and 57 weeks after the onset of diet. During sample collection mice were individually housed and fecal samples were collected in sterilized 1.5 mL tubes then immediately transferred to storage at −80 C until analysis. The sample prep and sequencing were performed as previously described (Kumar et al., 2014). QIIME2 was used for data analysis of the FASTQ files. DADA2 was the denoising algorithm used to generate the ASV tables. Alpha and Beta diversity metrices were analyzed using QIIME2 and rarefaction was done. LEfSe (Segata et al., 2011) was used for differential abundance analysis through the Galaxy module. Graphs were generated through R. phyloseq and vegan packages were used in R for microbiome analysis and Beta Diversity graphs.

### 2.6 Statistical analysis

Statistical analysis on microbiome data was carried out as described above. For all other experiments, two groups were compared using the two‐tailed student's *t* test with the welch correction applied where appropriate. Several groups were compared by two‐way ANOVA with post‐hoc testing carried out when significant main effects were detected. A two‐way PERMANOVA was carried out to Beta Diversity metrices. A p‐value <0.05 was considered statistically significant. All statistical analysis was performed using the R programming language, GraphPad Prism (8.4.2) and QIIME2.

## 3 RESULTS

We performed long‐term MR to examine its effects on the metabolism and gut microbiome in our experimental mice.

### 3.1 Long‐term MR diet feeding leads to body composition and metabolic changes

**FIGURE 1:**
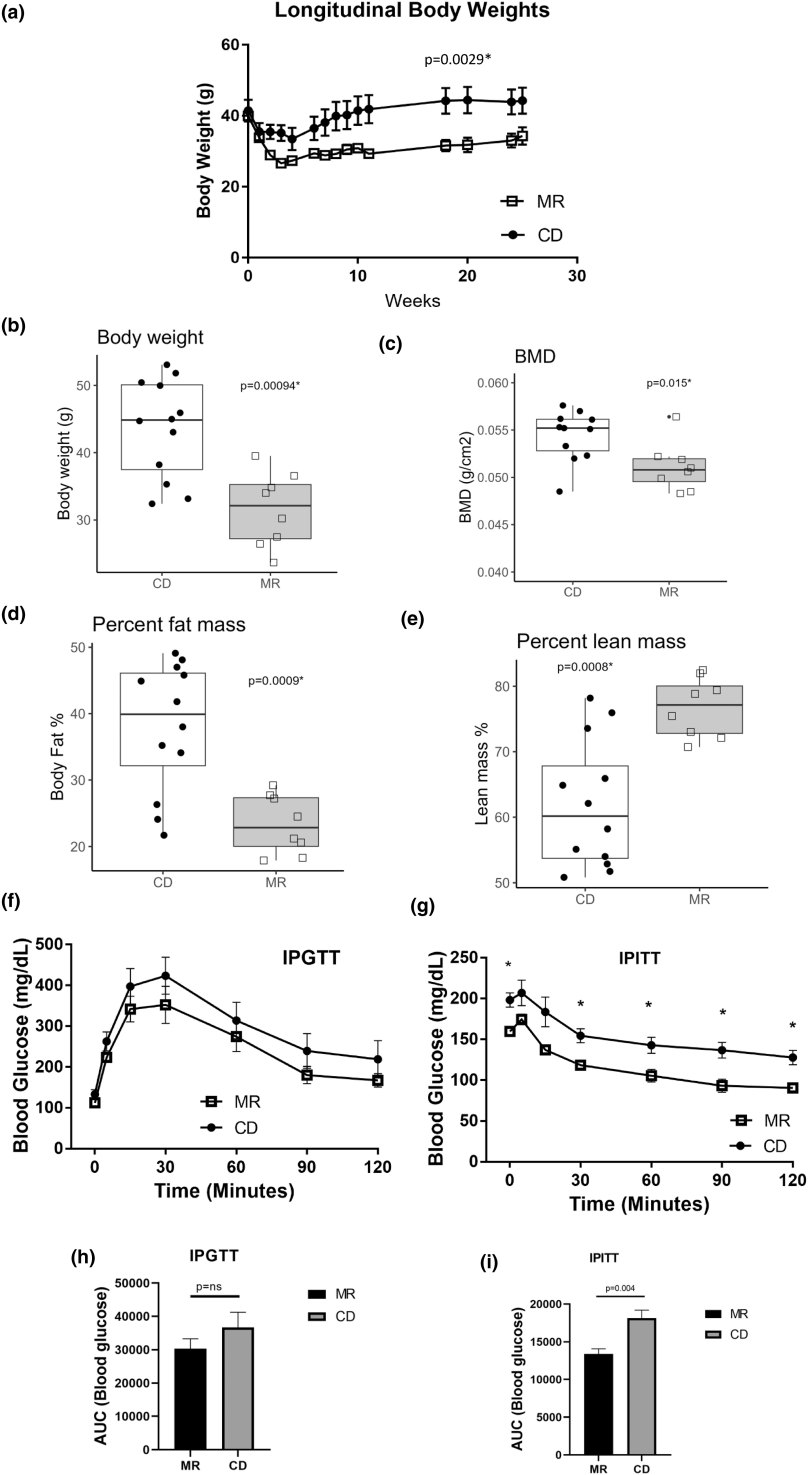
Weekly body weight and body composition parameters of MR and CD mice (a) Body weights of MR and CD mice from the start of diet till 6 months on diet. Body composition parameters of the MR and CD mice measured with DXA at 18 months of age and 48 weeks on diet (b–e). Body weight (b), BMD (c), Fat percent (d), lean percent (e). Intraperitoneal Glucose tolerance test (GTT) (f). MR and CD mice were fasted overnight for 16 h and injected with 1 g of glucose/kg body weight and insulin tolerance tests. Blood glucose measurements were taken the following 2 h. Intraperitoneal Insulin tolerance test (IPITT) (g) MR and CD mice were fasted for 4 h and injected with 1 IU of insulin/kg body weight. Blood glucose measurements were taken for the next 2 . Area under the curve analysis of IPGTT (h) and IPITT (i). Data presented as mean ± SEM for bar plots and line plots. Statistical comparison done using two‐tailed *t* test. *N* = 6 for body weight and food data, *N* = 8–12 for body composition, *N* = 6–7 for IPGTT and IPTTT.

Weekly body weight (Figure 1a) was collected during the initial period of diet change. Both groups of animals had similar body weights at the beginning of the experimental diet regime. In the first 2 weeks of diet onset, both MR and control diet (CD) mice lost body weight and the CD group regained body weight in the following weeks. The body weights of the MR group, as expected from previous studies, remained lower compared to the CD group (AUC *p* = 0.0029) (Figure 1a). Body composition was measured using DXA 48 weeks after MR feeding began (18 months of age). At this time, the MR mice had reduced body weight compared to the CD mice (*p* = 0.0009, Figure 1b). This reduction in body weight coincided with reductions in BMD and percent fat mass, and an increase in percent lean mass (*p* = 0.0148, *p* = 0.0009, and *p* = 0.0008 respectively (Figure 1c–e). Consistent with previous studies in different strains (Lees et al., 2017; Wanders et al., 2018), long term MR continued to limit fat accumulation in the experimental animals and this resulted in an overall increase in percent lean mass of the animals. We performed an IP glucose tolerance test (IPGTT) to assess glucose homeostasis in overnight (20‐h) fasted MR and CD mice at 12 months of age (24 weeks after diet onset). There were no changes in glucose tolerance between the MR and CD groups at any time point (Figure 1f, h). Next, we performed an IP insulin tolerance test (IPITT) to assess insulin sensitivity in 4‐h fasted MR and CD mice at 12 months of age (24 weeks after diet onset). As expected, the MR mice had significantly reduced blood glucose levels at all time points except at 5 and 15 min indicating an increase in insulin sensitivity (Figure 1g, i) (*p* < 0.05).

### 3.2 Long‐term MR diet feeding leads to increase in RER, metabolic rate, GOX but not FOX

**FIGURE 2:**
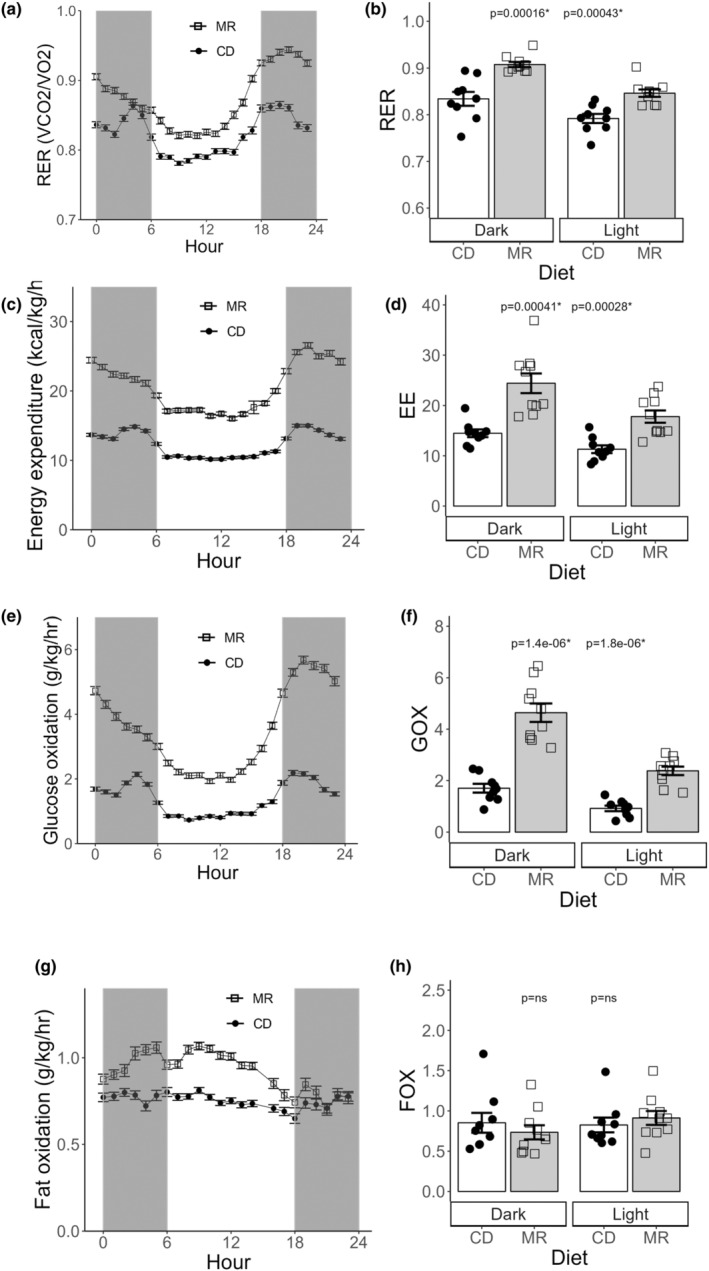
Respiratory exchange ratio (RER), Energy expenditure (EE), Glucose oxidation (GOX) and Fat oxidation (FOX) measured using indirect calorimetry in MR and CD mice. RER, EE, Glucose and Fat oxidation recordings averaged into a single day format split by light and dark cycles are plotted (a,c,e,g). RER, EE, Glucose and Fat oxidation recordings measured on light (b,d,f,h) and dark cycle averaged for individual animals. Data presented as mean ± SEM. Statistical comparison done using two‐tailed *t* test on the mean of the light and dark cycle values. *N* = 9–10 for Indirect Calorimetry.

To study the effect of long‐term MR diet feeding on the respiratory parameters of mice we performed extensive Indirect calorimetry experimentation to measure O2 consumption (VO2) and carbon dioxide production (VCO2) recordings. RER, EE, GOX, and FOX values were calculated as described in the methods section. RER (or Respiratory Quotient) is a metric which gives us an indication on what fuel source is being metabolized for energy. We have seen from various studies conducted before in mice and rats that MR increases RER of the animals (Plaisance et al., 2010). Consistent with previous studies, our long‐term MR mice had a significantly greater RER compared to CD mice during both the light (*p* = 0.0004) and dark cycles (*p* = 0.0001) of indirect calorimetry experiments (Figure 2a, b), indicating greater carbohydrate utilization relative to lipids in the MR mice. Previous studies have also shown us that MR significantly increases EE (Plaisance et al., 2010; Hasek et al., 2010). Here as expected, the MR mice had significantly increased EE compared to CD mice in both the light (*p* = 0.0004) and dark cycles (*p* = 0.0002), with the difference between the groups being notably more dramatic during the dark cycle (Figure 2g, h). The GOX and FOX rate of MR mice have not been previously reported in the literature using the indirect calorimetry method. In our study, we found that the GOX values (Figure 2c, d) of the MR mice were higher than that of the CD mice in both the light (*p* = 1.8e‐06) and dark cycle (*p* = 1.4e‐06) while no difference was observed in the FOX rates (Figure 2e, f) between the two groups.

### 3.3 Age has a significant effect on alpha and beta diversity metrices but not diet

**FIGURE 3:**
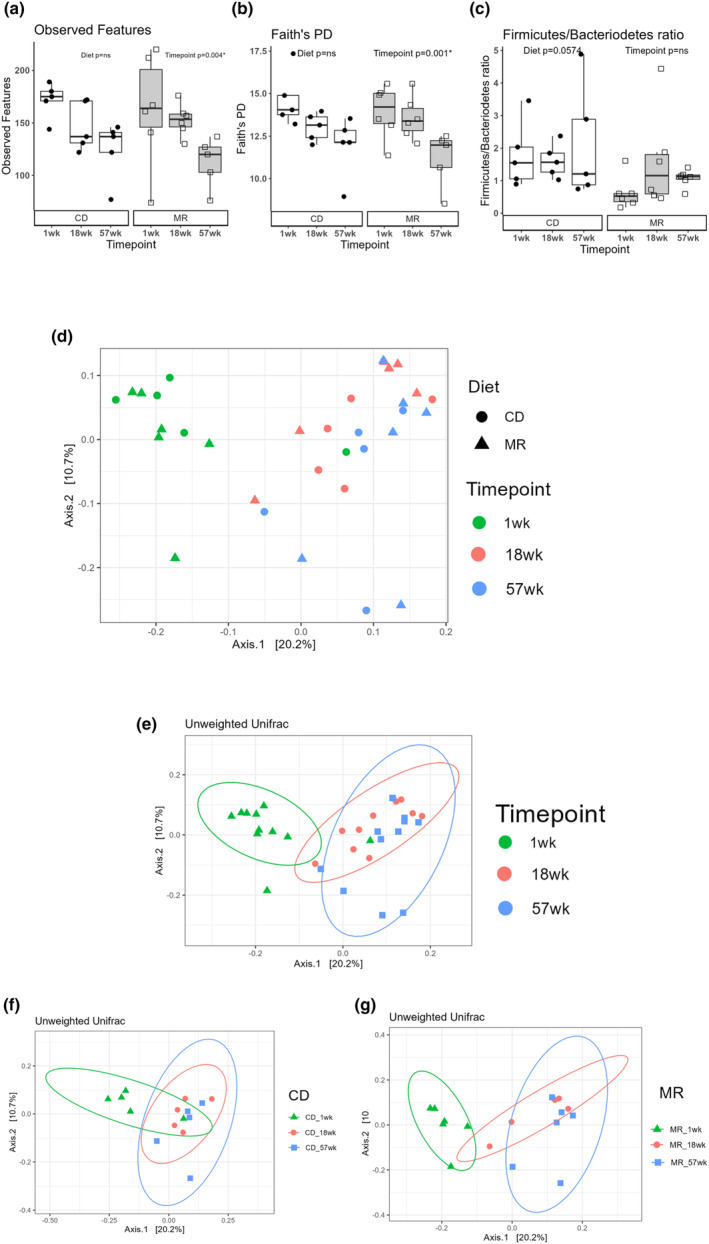
Diversity and Richness of the Gut Microbial communities. Faith's phylogenetic diversity (PD) (a) and Observed Features (b) for the MR and CD mice at 1 week, 18 weeks and 57 weeks after diet onset. *Firmicutes* and *Bacteriodetes* ratio (F:B ratio) (c) calculated by dividing the detected ASV's in the phylum *Firmicutes* and *Bacteriodetes* for the MR and CD mice at 1 week, 18 weeks and 57 weeks after diet onset. Principal Coordinates analysis (PCoA) plot of Unweighted Unifrac distances for all the groups (d). PCoA plot stratified for Age variable (e), and the two diet groups (f,g). Statistical comparison done using a two‐way ANOVA for Faith's PD, Observed Features and F:B ratio. Post‐hoc analysis was carried out using Tukey's HSD test. A two‐way PERMANOVA was used to analyze the Unweighted Unifrac distances. *N* = 5–6 per group.

16S rRNA sequencing was performed on samples collected from the experimental mice collected at three time points: 1, 18, and 57 weeks after diet onset. We calculated alpha and beta diversity metrics to analyze the richness and evenness of gut microbial communities. Alpha diversity gives us information about diversity within a sample and beta diversity gives us information about the similarity or dissimilarity of the gut microbial communities. Faith's phylogenetic diversity and Observed features were the two alpha diversity metrics calculated. The timepoint variable had a significant effect on both Observed Features (*p* = 0.004, Figure 3a) and phylogenetic diversity (*p* = 0.001, Figure 3b). The mean of the alpha diversity metrics decreased with age with the lowest values at 57 weeks. Although timepoint had an overall effect, post hoc pairwise comparisons did not detect any changes between individual groups. The Diet variable did not have a significant effect on phylogenetic diversity or observed features. The Firmicutes to Bacteroidetes ratio (F/B) has been associated with various aspects of health and metabolism, and also linked with obesity (Magne et al., 2020). We calculated the Firmicutes to Bacteroidetes ratio (F/B) to check for differences between the groups. We found Diet had a strong trend towards reducing the F/B ratio (*p* = 0.0574) but it did not reach statistical significance (Figure 3c). Timepoint did not have any effect on the F/B ratio although most other parameters changed with age.

To assess the similarity or dissimilarity between microbial communities we analyzed beta diversity metrics using Unweighted Unifrac distance. Principal coordinates analysis (PCoA) plots were visualized using the calculated Unweighted Unifrac distances (Figure 3d–g). A 2way PERMANOVA was performed on the distance between the groups to analyze the group differences. It showed that the Timepoint variable had a significant effect (*p* = 9.999e‐05) in altering the microbial community structure while Diet did not have a significant effect (*p* = 0.0834, Figure 3d). There was a striking change in the community structure between 1‐week and 57‐week groups in both the MR (Figure 3f, *p* = 0.015) and CD mice (Figure 3g, *p* = 0.045) and they were significantly different.

### 3.4 Most dietary changes in bacterial taxa are detected at 1 and 18 weeks and not at 57 weeks

**FIGURE 4:**
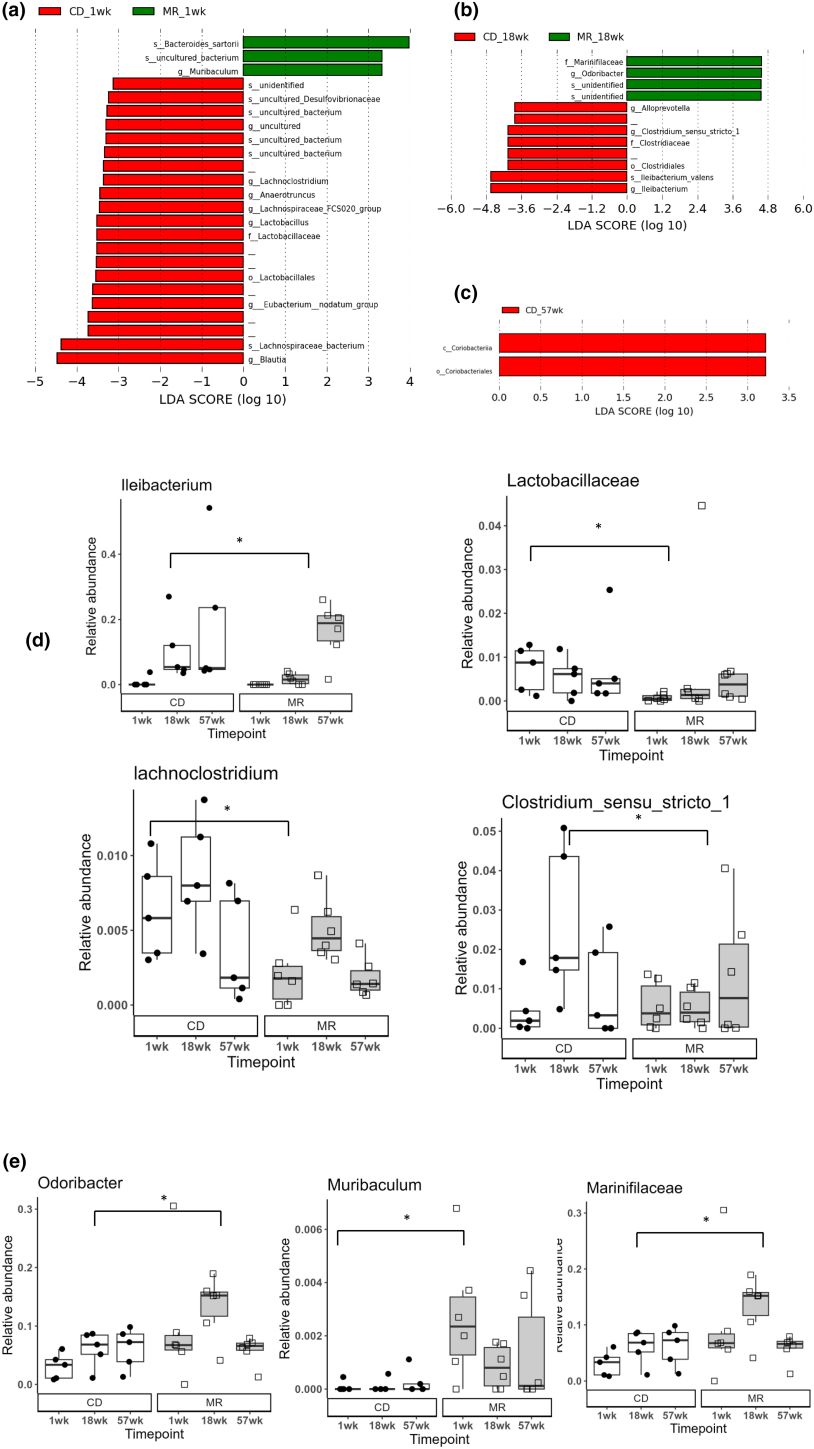
Gut Microbiota changes due to diet. LEfSe used to determine the microbial taxa that are increasing or decreasing in MR or CD mice at 1 week (a),18 week (b) and 57 weeks (c) after diet onset visualized using LDA histograms. A select few differentially abundant taxa due to diet at 1 or 18 week of diet onset (d,e). LDA Histograms represent the differentially abundant taxa in each group at each timepoint, green bars are enriched in MR and red bars are enriched in CD group. Bars without taxa name indicate they remain unclassified after SILVA database classification. Statistical comparison done using LEfSe, * indicates statistical significance with LDA score >3 or < −3. Blank lines on the LDA Histogram indicate bacterial taxa which remain unclassified. *N* = 5–6 per group.

Differential abundance analysis to detect dietary changes was performed using LEfSe (Segata et al., 2011) at 1, 18, and 57 weeks after diet onset. The 1‐week group (Figure 4a) had the highest number of differentially abundant taxa followed by the 18‐week group (Figure 4b) and 57‐week group (Figure 4c). Some bacterial taxa which were found to be changing *were Ileibacterium, Lactobacillaceae*, *Clostridium_sensu_stricto_1*, and *Lachnoclostridium* decreased in MR mice at 1 or 18 weeks while *Odoribacter*, *Muribaculum*, and *Marinifilaceae* were found to be increasing in MR mice at 1 or 18 weeks (Figure 4d,e).

### 3.5 Increasing age leads to significant changes in bacterial taxa in both MR and CD mice

**FIGURE 5:**
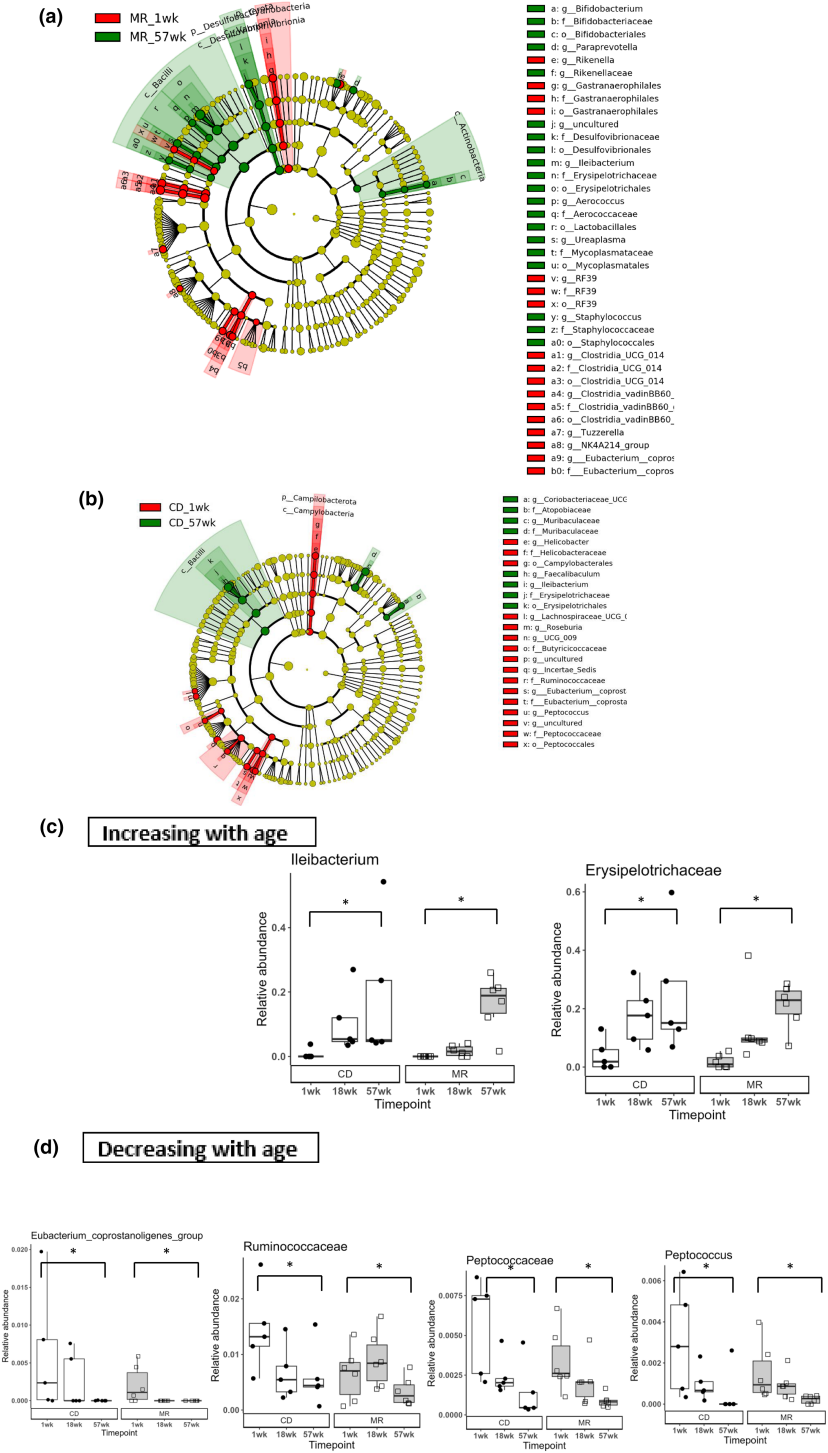
Age‐related changes to the gut microbiota and conserved changes between the diet groups. LEfSe used to calculate differential abundance. Cladogram representing the differentially abundant taxa between 1 week and 57 week animals in the MR group (a) and CD group (b). Conserved aging‐associated changes between the MR and CD mice. Taxa increasing (c) or decreasing (d) due to age in both MR and CD mice. Each cladogram (a,b) represents all taxa detected at >0.1%, shown at the Kingdom phylogenetic level through the genus level. A yellow circle depicts taxa present, but not enriched. Red circles are enriched in aging, and green enriched in young animals. The size of the circle corresponds to the population of each taxon. Statistical comparison done using LEfSe, * indicates statistical significance with LDA score >3 or < −3. *N* = 5–6 per group.

Age‐related changes in bacterial taxa in the MR (Figure 5a) and CD mice (Figure 5b) were identified using LEfSe. In the 1 week vs 57 week timepoint comparison, we found more age‐related changes were detected in the MR mice compared to the CD mice. Conserved age‐related changes between both MR and CD groups were observed. *Ileibacterium* and *Eryspelotrichceae* were found to be increasing with age in both the diet groups (Figure 5c) while *Eubacterium_coprostanoligenes_group, Ruminococcae, Peptococcae*, and *Peptococcus* were found to be decreasing with age in both the diet groups (Figure 5d).

### 3.6 Specific age‐related family level changes occurring in MR or CD mice

**FIGURE 6:**
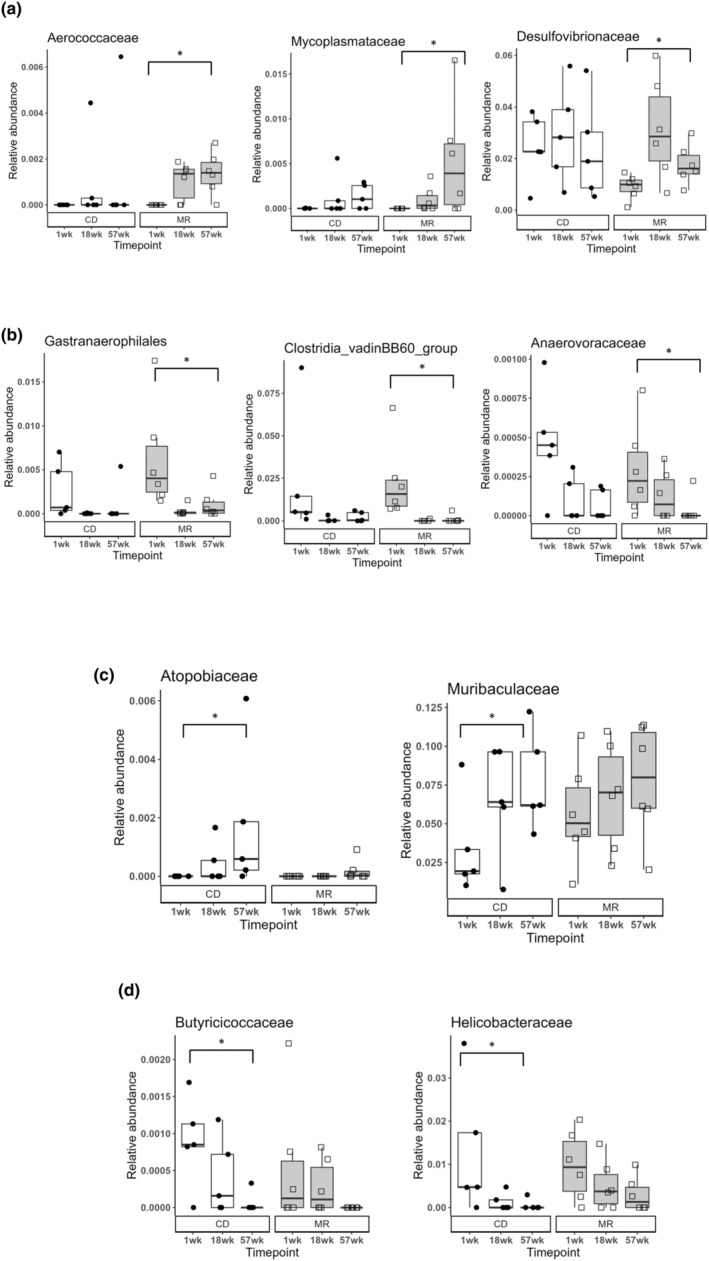
Age‐related changes to the gut microbiota and distinct changes between the diet groups. Box plots of select differentially abundant bacterial taxa found increasing (a) or decreasing (b) with age in MR mice at the Family level. Bacterial taxa found increasing (c) or decreasing (d) with age in CD mice at the Family level. Statistical comparison done using LEfSe, * indicates statistical significance with LDA score >3 or < −3. *N* = 5–6 per group.

Apart from the conserved age‐related changes in both the diet groups, some changes were detected in either only the MR or only the CD group*. Aerococcaceae, Mycoplasmataceae. Desulfovibrionaceae* were increasing with age in the MR mice (Figure 6a) while *Clostridia_vadinBB60_group, Gastranaerophilales*, and *Anaerovoracaceae* were decreasing with age in the MR mice (Figure 6b). *Atopobiaceae* and *Muribaculaceae* were increasing with age in the CD mice (Figure 6c) while *Helicobacteraceae* and *Butyricicoccaceae* were decreasing with age in the CD mice (Figure 6d).

## 4 DISCUSSION

The advancement of sequencing technologies over the past two decades has allowed for extensive study of the gut microbiome composition in humans and laboratory animals. Gut microbiota has been associated with a lot of disease states, leading to the hypothesis that it may play a causal role in diseases. In our study, we profiled the microbiome changes with a MR diet in mice starting in adulthood at 6 months through 20 months of age. We found that a long‐term MR surprisingly has a minimal effect on the microbiome while we found age to have a large effect on the microbiota in our current study. We observed short‐term changes with a methionine‐restricted diet when we sampled at 1 week and 18 weeks after diet onset but after 57 weeks the microbiota stabilized and most of the bacterial taxa changes disappeared.

As observed from earlier studies we found that MR caused decreases in body weight and adiposity along with increased insulin sensitivity (Sun et al., 2009) (Plaisance et al., 2010) (Stone et al., 2014) in our experimental mice. There was also increase in RER and EE consistent with the published literature (Plaisance et al., 2010). BMD was reduced in our MR mice similar to previous MR studies (Li et al., 2016)and CR was also shown to reduce BMD (Villareal et al., 2006). This may be a feature of body‐weight reducing dietary regimens which improve metabolic parameters. Glucose tolerance measured by GTT showed no changes in our study similar to other studies (Castaño‐Martinez et al., 2019; Wang et al., 2020) but improved glucose tolerance was found in other studies (Lees et al., 2017). This may be due to factors like strain of mice, age, time/duration of diet regimen and dosage and fasting time. Our indirect calorimetry results were similar to previous published reports of MR (Plaisance et al., 2010) significantly increasing EE of mice and rats and also increasing RER. Our results were different from other long lived models like growth‐hormone releasing hormone knockout (GHRH‐KO) mice(GHRH‐KO) and CR mice. GHRH‐KO mice (Icyuz et al., 2020) have lower EE overall and lower RER in light cycle while CR mice (Schloesser et al., 2015) have lower EE. We report for the first‐time GOX and FOX rates in the MR mice using the indirect calorimetry procedure.

CR, protein restriction, and MR (Solon‐Biet et al., 2014; Sun et al., 2009; Weindruch et al., 1986) dietary regimens have been shown to increase lifespan and drive metabolic changes in various model organisms. Recently there have been studies (Pak et al., 2019; Wallis et al., 2020) investigating the contribution of gut microbiota to these observations. The delayed aging and lifespan extension due to these interventions have been hypothesized to occur due to at least partly similar pathways (Sun et al., 2009). There is evidence that some metabolic effects of CR are mediated by the microbiome. Microbiota transplantation experiments from calorie‐restricted mice to germ‐free mice recapitulated some effects observed following calorie‐restriction diets (Fabbiano et al., 2018) while calorie restriction on microbiome‐depleted animals was found to have differing and smaller effects on metabolism (Wang et al., 2018). In the context of PR diets however, it was seen that their metabolic effects were unlikely to be mediated by the microbiome as a PR diet worked just as well in mice with antibiotic‐mediated microbiome ablation (Pak et al., 2019). Interestingly, it was recently found that the FGF‐21 response to protein restriction is mediated by the microbiota (Martin et al., 2021) with protein restriction in conventional mice causing a dramatic increase in plasma FGF‐21 and mRNA levels while these effects were blunted in germ‐free mice.

In the past few years, there have been several reports examining the effects of MR in mice. Two of these studies were in high‐fat diet‐fed mice, one in young mice, one in old mice, and another in D‐galactose‐fed aging mice. But all these studies have focused on short‐term changes ranging from 10 weeks to 5 months of dietary intervention. Ours is the first study focused on feeding effects on the microbiome. In the published literature so far, there has been a general trend in the differences observed with significant changes in beta diversity metrics for most cases but not much changes in alpha diversity metrics. SAAR (Sulphur amino acid restriction) performed in 18‐week BL/6 mice for 10 weeks found no significant change in beta diversity between the SAAR and CD group and a few changes in bacterial taxa between those two groups namely *Clostridiaceae* and 94OTU972 (*Clostridium paraputrificum*) (Nichenametla et al., 2021). In a study of MR on aged mice, it was observed that the Simpson index was increased while no changes were seen in the other 4 alpha diversity parameters with clustering between groups observed for beta diversity (Ren et al., 2021). In the high‐fat diet studies combined with methionine restriction, changes were seen for some alpha diversity parameters and the beta diversity was significantly different between groups (Wu et al., 2020; Yang et al., 2019). In our study, we surprisingly did not detect any changes in alpha or beta diversity for the Diet variable at any of the time points. The age of the mouse had a significant effect, with the Timepoint variable being significantly different for both alpha and beta diversity. We detected several bacterial taxa changes due to Diet at 1 week and 18 weeks but surprisingly not at 57 weeks. In our current study, we found alpha diversity metrics decreased with age, similar to our earlier published report where we saw alpha diversity decrease with age in Fischer344 rats (Nagarajan et al., 2023). However, others investigating the mouse microbiome did not report any changes in diversity with age (Binyamin et al., 2020). Beta diversity changes and modulation of community structure with age have been reported in another mice study (Binyamin et al., 2020) and our earlier rat study (Nagarajan et al., 2023; Srivastava et al., 2023). This is further evidence that the gut microbiota changes significantly with age. The F/B ratio did not differ with age in our current study or our previous study with F344 rats (Nagarajan et al., 2023). The work of Binyamin and colleagues conflicts with ours, however, as they have reported an increased F/B ratio with advanced age (Binyamin et al., 2020). The F/B ratio of MR mice under a high‐fat diet was found to be increased compared to normal mice under a high‐fat diet, although in our study we found a general trend toward decrease.

Some notable bacterial taxa changes we found was genus *Ileibacterium* which decreased in MR mice at 16 weeks after diet onset and it was also increasing with age at 57 weeks in both the diet groups. *Ileibacterium* belongs to the family *Erysipelotrichaceae* and it was increasing with age in our current study and our previous study in both WT and TgF344‐AD rats. Similar results have been observed in other published reports. *Ruminococcaceae*, a prominent butyrate producer (Vital et al., 2017) was another significant taxa change that decreased with age in both our diet groups. Low levels of *Ruminococcaceae* have been associated with antibiotic‐associated diarrhea (AAD) (Gu et al., 2021). Another study found an inverse correlation between fibrosis severity and decreased *Ruminococcaceae* abundance, with heightened fibrosis being associated with lower abundance (Lee et al., 2020). A prebiotic supplementation study in older frail people resulted in a family *Ruminococcaceae* increase along with other changes (Tran et al., 2019). Two human trials, one with peanut feeding (Sapp et al., 2022)and the other with herbs and spices added to the diet (Petersen et al., 2022), found that *Ruminsococcaceae* was increased in both cases. All these studies point to the family *Ruminococcaceae* being associated with healthy states and its decrease was observed with some negative phenotypes. We found the genus *Roseburia* to also decrease with age. Species in the genus *Roseburia* have been associated with numerous health benefits recently (Nie et al., 2021). In our study we found that long term MR diet did not significantly alter the microbiota compared to short term feeding and that the microbiome does not play a role in MR diet's influence on metabolism in lab mice. One of the top candidates of how MR diet influences metabolism may be through FGF‐21 signaling. MR increases FGF21 levels and it is required for many metabolic changes occurring in MR (Fang et al., 2021). Other possible factors may be through inhibiting the GH/IGF1 axis, as MR mice have lower IGF‐1 levels (Plummer & Johnson, 2022). Longevity studies has also shown that MR on GH‐deficient mice does not further extend their lifespan (Brown‐Borg et al., 2014). This may be because the lifespan extending effect of MR and GH‐deficient mice may occur due to similar pathways. Overall, we found that aging caused significant changes in bacterial taxa and some of those changes were specific to either MR or CD mice.

## AUTHOR CONTRIBUTIONS

Akash Nagarajan conducted experiment, helped formulate experiment design, analyzed data, and drafted the manuscript. Alexander Tate Lasher assisted in data analysis and manuscript drafting. Casey D Morrow helped with microbiome sample processing. Liou Y. Sun conceived the study, oversaw overall direction, and secured funding. All authors edited the manuscript and provided critical feedback that helped shape the research, analysis, and manuscript.

## FUNDING INFORMATION

This work was supported in part by the National Institute on Aging grants AG082327, AG057734, and AG050225 (L. Y. S.).

## CONFLICT OF INTEREST STATEMENT

All the contributing authors declared no conflicts of interest.

## Data Availability

The data supporting the findings of this study are available from the corresponding author upon reasonable request.
